# Impact of Different Cleaning Protocols on the Optical and Morphological Properties of 3D-Printed Aligners After *In Vitro* Aging

**DOI:** 10.1055/s-0045-1813031

**Published:** 2025-12-18

**Authors:** Ibrahim Yawoz Abdulkarim, Sara M. Al-Mashhadany

**Affiliations:** 1 Department of Orthodontic, College of Dentistry, University of Baghdad, Baghdad, Iraq

**Keywords:** 3D printing, AFM, artificial aging, cleaning methods, clear aligners

## Abstract

**Objectives:**

In-house printing of clear aligners (CAs) may represent a new revolution in CA therapy, providing a solution for the several limitations associated with indirect manufacturing. This study compared the changes in surface roughness and light transmittance (T%) of directly printed (DP) and thermoformed (TF) CAs after exposure to *in vitro* aging and different cleaning methods.

**Materials and Methods:**

A total of 48 aligners were fabricated using either multilayer thermoplastic polyurethane (CA Pro + , Scheu Dental; Iserlohn, Germany) or direct three-dimensional-printed resin (Tera Harz TC-85, Graphy; Seoul, Korea). CAs were aged *in vitro* (thermocycling and artificial saliva) and cleaned for 14 days with the following methods: Polident tablets, an electric toothbrush, Pril dish soap, an ultrasonic cleaning machine, and distilled water. Subsequently, alterations in surface roughness and T% were investigated using atomic force microscopy and spectrophotometry, respectively. This study was conducted from January to July 2025 at the Department of Orthodontics, College of Dentistry, University of Baghdad, Baghdad, Iraq, with additional laboratory work at the University of Technology/Ministry of Science and Technology.

**Statistical Analysis:**

Data analysis included descriptive statistics, independent *t* -tests, one-way analysis of variance, and post hoc tests (Tukey's honestly significant difference or Games–Howell).

**Results:**

Compared with the DP aligners, the TF aligners presented greater T% under all conditions, with the highest T% in the as-received condition. The TF and DP aligners showed the lowest T% in ultrasonic cleaning and control conditions, respectively. When excluding the as-received group, most cleaning methods showed statistically significant differences in T%. The surface roughness of the TF aligners was consistently lower than that of the DP aligners, with significant differences observed between the Polident and control groups.

**Conclusion:**

Cleaning methods altered the optical and morphological properties of both aligners. Customized cleaning methods are required for CAs.

## Introduction

The primary goal of orthodontic treatment is to improve the esthetic and function of malocclusion. 1 The rising demand for esthetic orthodontic treatments, alongside the development of computer-aided design and manufacturing technology, has allowed the incorporation of clear aligners (CAs) into available treatment options. 2 CAs provide a discreet and comfortable alternative to conventional orthodontic methods that utilize numerous brackets, demonstrating comparable efficacy in mild to moderate cases of malocclusion. 3 4 Their advantages include enhanced esthetics, removability, oral hygiene maintenance, and shorter therapeutic duration. 5 6 However, the surface roughness of the CAs can influence plaque accumulation, biofilm adhesion, loss of transparency, and discoloration. 7

In-house three-dimensional (3D) printing of CAs has emerged as a new revolution in orthodontics, 8 addressing limitations such as geometric discrepancies, thermal distortions, and irregular fitting or layer thickness. 9 10 11 Tera Harz TC-85 Direct Aligner Clear (DAC) resin—a photopolymerizable polyester-urethane polymer, possesses biocompatible Class IIa CE certification and is approved by both the European Commission and the Korean Food and Drug Administration, facilitating the direct 3D printing of orthodontic CAs since 2019. Despite its potential benefits, the properties of this new material are poorly characterized. 12

Maintaining the esthetic appearance and surface integrity of thermoplastic materials is a key concern for patients and orthodontists, due to their tendency of stain absorption. 13 Although clinical guidelines recommend removing CAs prior to eating or drinking, patient compliance is evident to be poor. 14 15

Several studies indicate that the accumulation of biofilm on aligner surfaces is affected by the properties of the material and the hygiene practices of patients. 16 17 The predominant cleaning methods are primarily mechanical or chemical. Considering the microbial challenges linked to aligner usage and the influence of surface texture on biofilm formation, the implementation of effective cleaning protocols is crucial for maintaining the integrity of thermoplastic and 3D-printed materials. 18 19 Peroxide-based cleaners effectively reduce microbial load without substantially altering the properties of the aligner, 20 21 22 their impact on light transmittance and surface characteristics when compared with other cleaning methods such as mechanical brushing, ultrasonic cleaning, and chemical disinfectants—including Pril dish soap and Polident tablets, remains inadequately investigated. While thermoformed (TF) aligners are widely researched, 3D-printed aligners embody an innovative technology characterized by distinct material surface qualities, which may restrict the applicability of current cleaning methods. Unlike TF aligners, 3D-printed aligners use photopolymerizable resins instead of thermoplastic sheets. Although visually similar, their unique material characteristics may affect their reaction to conventional cleaning techniques recommended by practitioners. Therefore, this study aimed to compare the effects of *in vitro* aging and the application of various cleaning methods on the surface roughness and light transmittance of directly printed (DP) and TF orthodontic aligners.

### Null Hypothesis

The surface roughness and light transmittance of the TF aligners before and after cleaning and aging do not differ from that of the DP aligners.

## Materials and Methods

### Study Design

This *in vitro* study was conducted between January and July 2025 at the Department of Orthodontics, College of Dentistry, University of Baghdad, Baghdad, Iraq, with supplementary laboratory work performed at the University of Technology/Ministry of Science and Technology.

### Materials

Two types of aligners were tested: (1) multilayer thermoplastic polyurethane (CA Pro + , Scheu Dental; Iserlohn, Germany) and (2) direct 3D-printed aligners (Tera Harz TC-85 DAC resin, Graphy, Seoul, Korea). Cleaning agents included the following: Polident cleanser tablets (Stafford-Miller, Dungarvan Co., Waterford, Ireland), an electric toothbrush Pro 300 (Oral-B; Braun, Germany), Pril dish soap (Henkel, Düsseldorf, Germany), an ultrasonic cleaner (Mingpinhui, China), and distilled water. Pril soap was measured at 0.3 g per six teeth using an analytical balance (JOANLAB, China).

### Sample Preparation and Cleaning Procedures

The sample size was determined using G*Power 3.1 (University of Düsseldorf, Germany), referencing prior studies, 23 24 requiring a minimum of four samples per group ( *α* = 0.05, power = 80%). A total of 48 aligners (24 per material) were allocated into six groups ( *n* = 4):

*- Group (P)* : Cleaned with a Polident tablet in 100 mL distilled water for 5 minutes daily.
*- Group (X)* : Mechanically brushed with an electric toothbrush and distilled water for 2 minutes daily.
*- Group (XP)* : Brushed using an electric toothbrush and a Pril soap solution (0.1 g soap + 2 mL distilled water) for 2 minutes daily, then rinsed.
*- Group (U)* : Cleaned ultrasonically in distilled water for 15 minutes daily.
*- Group (Ctr)* : *In vitro* aging only.
*- Group (AR)* : As-received (dry condition) for baseline reference.

These cleaning tools were selected for their clinical relevance, as they represent common, widely available mechanical and chemical protocols recommended in orthodontic practice.

### Aligner Fabrication

A standardized typodont model was scanned using Aoralscan 2 (Shining 3D, China), saved in a standard tessellation language file format, and printed using S-2 black resin (Shining 3D). CA Pro+ sheets were then TF using Ministar S (Scheu-Dental GmbH, Germany) onto the model per manufacturer specifications (220°C, 30 seconds heating, 60 seconds cooling, 7 seconds evacuation, 4 bar pressure). For testing, each aligner was sectioned lingually from canine to canine to expose the labial surface of the maxillary right central incisor, which was selected for its relatively flat geometry.

An AccuFab-L4K 3D printer was used to manufacture the DP aligners using Tera Harz TC-85 DAC resin. Models were oriented with the posterior section angled 45 degrees toward the build platform and printed at a layer thickness of 50 µm. The final thickness was chosen as 0.5 mm to account for the anticipated reduction in CA Pro+ (0.75 mm) after thermoforming. To obtain the finest details for each model, the layer thickness was set at 50 µm. Upon completion of the printing process, the aligners were detached from the build platform using a soft scraper.

Excess uncured resin was removed by centrifugation (6 minutes at 600 rotations per minute using a centrifuge machine [GRAPHY, Korea]), followed by ultrasonic rinsing (3 minutes with an ultrasonic cleaner [Silvercrest, Germany]). The aligners were air-dried for 5 minutes on paper towels and cured at ultraviolet (UV) power level 2 for 20 minutes under nitrogen using a Tera Harz Cure THC 2 UV curing machine (Graphy, Seoul, Korea). This device is equipped with a built-in nitrogen generator and designed for postprocessing with elevated UV energy and irradiance to achieve expedited curing durations and enhance the mechanical properties of printed aligners.

### Aging of Aligners

Initially, aligners were immersed in distilled water for 24 hours, then thermocycled for 500 cycles (5°C and 55°C; 20-second dwell, 5-second transfer) per ISO/TS 11405:2015.25. 25 Subsequently, they were stored individually in artificial saliva and incubated at 37°C for 14 days.

### Testing

The light transmittance (T%) was assessed and measured using a UV/visible spectrophotometer (LAMBDA 365, PerkinElmer Inc., United States). Surface topography was evaluated at the nanoscale level using atomic force microscopy (AFM). It was utilized in tapping mode, which allowed intermittent contact between the probe tip and the surface of the sample.

### Statistical Analysis

The data were statistically analyzed using the Statistical Package for Social Sciences (IBM SPSS software, Armonk, United States) version 26.0. The normality of the distribution and homogeneity of variances among groups were assessed using the Shapiro–Wilk and Levene's tests. Descriptive statistics included means, standard deviations (SDs), and statistical tables and figures. Inferential statistics for parametric data included independent *t* -tests, one-way analysis of variance (ANOVA), and Tukey's honestly significant difference (HSD) post hoc tests where significant differences were detected. Welch's test and the Games–Howell post hoc test were used when the assumption of homogeneity of variances was violated.

## Results

The Shapiro–Wilk and Levene's tests confirmed normality and homogeneity assumptions. Means, SDs, and comparison of T% between the TF and DP aligners, as well as the ANOVA results across different cleaning conditions, are presented in Table 1 . Under all conditions, the T% mean values for the TF aligners were greater than those for the DP aligners. For the TF aligners, the highest T% was observed under the AR group (95.79%), whereas the lowest was observed under the U condition (41.95%). In contrast, the DP aligners presented the highest T% under AR (87.26%) and the lowest T% under Ctr (10.49%), and the difference was significant between TF and DP in all cleaning conditions (P, *p* = 0.000; X, *p* = 0.002; XP, *p* = 0.000; U, *p* = 0.006; Ctr, *p* = 0.005), except for the AR group ( *p* = 0.067), where there was no significant difference between the two materials.

**Table 1:** Mean values, standard deviations, and comparison of T% between TF and DP aligners and ANOVA test among different cleaning conditions

Conditions		TF		DP		Comparison between TF and DP	
		Mean	SD	Mean	SD	Independent sample *t* -test	*p-* Value
P		71.87	± 8.71	18.9	± 9.54	8.199	0.000 a
X		63.91	± 6.45	39	± 6.84	5.296	0.002 a
XP		75.22	± 3.12	25.15	± 4.51	18.275	0.000 a
U		41.95	± 4.20	20.97	± 9.07	4.2	0.006 a
Ctr		52.37	± 19.23	10.49	± 4.48	4.242	0.005 a
AR		95.79	± 1.94	87.26	± 7.38	2.235	0.067
ANOVA	*F* -test	16.4		59.27			
	*p* -value	0.00 a		0.00 a			

Abbreviations: ANOVA, analysis of variance; AR, as received; Ctr, control; DP, directly printed; P, Polident; SD, standard deviation; TF, thermoformed; U, ultrasonic cleaning; X, brushing; XP, brushing + Pril soap.

a Highly significant difference ( *p* ≤ 0.01).

One-way ANOVA test revealed highly significant differences ( *p* = 0.00) among cleaning conditions for TF and DP aligners. For the TF aligners, post hoc Tukey's HSD tests revealed a significant difference in T% between the AR group and other conditions, except for the XP group. The U group also differed significantly from other groups, except for the Ctr group ( *p* = 0.618). For the DP aligners, the AR group differed significantly from all other conditions, and the X group differed from all except the XP group ( *p* = 0.123), as shown in Table 2 . The results suggest that cleaning method, aging, and material type may have a visible impact on esthetics during aligner wear.

**Table 2:** Post hoc Tukey's HSD test for multiple comparisons among groups

Condition		TF		DP	
		Mean difference	*p* -Value	Mean difference	*p* -Value
AR	P	−23.92	0.020 **a**	−68.36	0.000 **b**
AR	X	−31.88	0.002 **b**	−48.26	0.000 **b**
AR	XP	−20.57	0.055	−62.11	0.000 **b**
AR	U	−53.84	0.000 **b**	−66.29	0.000 **b**
AR	Ctr	−43.42	0.000 **b**	−76.77	0.000 **b**
Ctr	P	19.5	0.075	8.4	0.584
Ctr	X	11.54	0.515	28.51	0.000 **b**
Ctr	XP	22.86	0.027 **a**	14.66	0.092
Ctr	U	−10.42	0.618	10.47	0.357
U	P	29.92	0.003 **b**	−2.07	0.998
U	X	21.96	0.036 **a**	18.04	0.025 **a**
U	XP	33.28	0.001 **b**	4.19	0.96
XP	P	−3.35	0.995	−6.26	0.821
XP	X	−11.31	0.536	13.85	0.123
X	P	7.96	0.826	−20.11	0.011 **b**

Abbreviations: AR, as received; Ctr, control; DP, directly printed; HSD, honestly significant difference; P, Polident; TF, thermoformed; U, ultrasonic cleaning; X, brushing; XP, brushing + Pril soap.

a Significant difference ( *p* < 0.05).

b Highly significant difference ( *p* ≤ 0.01).

The Shapiro–Wilk test indicated that the surface roughness data (Sa values) were normally distributed under most conditions, as the *p* -values exceeded 0.05. Levene's test revealed a violation of the homogeneity of variance assumption for the TF groups ( *p* = 0.018). The means, SDs, and comparison of the arithmetic mean height (Sa) values for the TF and DP aligners, along with the ANOVA test comparison among the different cleaning conditions, are shown in Table 3 .

**Table 3:** Mean values, standard deviations, and comparison of the Sa values between TF and DP aligners and ANOVA test comparison among different cleaning conditions

Conditions		TF		DP		Comparison	
		Mean	SD	Mean	SD	*t* -Test	*p* -Value
P		10.65	± 1.49	56.29	± 18.20	−4.998	0.002 a
X		17.39	± 1.95	40.86	± 19.20	−2.432	0.051
XP		26.86	± 18.76	33.27	± 15.05	−0.532	0.614
U		42.33	± 33.48	32.12	± 7.97	0.593	0.575
Ctr		23.16	± 13.15	70.99	± 18.12	−4.273	0.005 a
AR		63.72	± 19.35	65.45	± 9.93	−0.159	0.879
ANOVA	*F* -test			4.75			
	*p* -value			0.006 a			
Welch test	*p* -value	0.003 a					

Abbreviations: ANOVA, analysis of variance; AR, as received; Ctr, control; DP, directly printed; P, Polident; SD, standard deviation; TF, thermoformed; U, ultrasonic cleaning; X, brushing; XP, brushing + Pril soap.

a Highly significant difference ( *p* ≤ 0.01).

Overall, for the TF samples, the mean nanoroughness (Sa) values ranged from 10.65 ± 1.49 nm in the P group to 63.72 ± 19.35 nm in the AR group. The greatest variability was observed in group U (SD = 33.48 nm). For the DP samples, the highest Sa value was recorded for the Ctr group (70.99 ± 18.12 nm), whereas the lowest value was recorded for the U group (32.12 ± 7.97 nm). Statistically significant differences were observed between the P (TF: 10.65 ± 1.49 nm; DP: 56.29 ± 18.20 nm; *p* = 0.002) and Ctr (TF: 23.16 ± 13.15 nm; DP: 70.99 ± 18.12 nm; *p* = 0.005) groups, with the DP materials showing significantly greater roughness values.

Welch and one-way ANOVA confirmed significant differences among cleaning conditions for TF ( *p* = 0.003) and DP ( *p* = 0.006) aligners, respectively. Games–Howell post hoc analysis for TF samples showed a statistically significant difference in Sa values between the P and X groups ( *p* = 0.014). For the DP samples, Tukey's HSD post hoc test revealed significant differences between the XP and Ctr groups ( *p* = 0.028) and between the U and Ctr groups ( *p* = 0.022), with the Ctr group exhibiting the highest Sa values, as shown in Table 4 . Although nanoroughness increased significantly among groups, values remained below the clinically relevant threshold of 0.20 µm.

**Table 4:** Games–Howell and Tukey's HSD post hoc tests for multiple comparisons among groups

Condition		TF		DP	
		Mean difference	*p* -Value	Mean difference	*p* -Value
AR	P	−53.07	0.054	−9.16	0.955
AR	X	−46.33	0.077	−24.59	0.259
AR	XP	−36.86	0.197	−32.19	0.076
AR	U	−21.39	0.86	−33.34	0.062
AR	Ctr	−40.57	0.099	5.54	0.995
Ctr	P	−12.51	0.528	−14.70	0.753
Ctr	X	−5.77	0.933	−30.13	0.108
Ctr	XP	3.71	0.999	−37.73	0.028 a
Ctr	U	19.17	0.873	−38.88	0.022 a
U	P	−31.68	0.53	24.18	0.274
U	X	−24.94	0.695	8.75	0.963
U	XP	−15.47	0.954	1.15	1.000
XP	P	−16.21	0.595	23.03	0.321
XP	X	−9.48	0.891	7.60	0.980
X	P	−6.74	0.014 b	15.43	0.715

Abbreviations: AR, as received; Ctr, control; DP, directly printed; HSD, honestly significant difference; P, Polident; TF, thermoformed; U, ultrasonic cleaning; X, brushing; XP, brushing + Pril soap.

a Significant difference ( *p* < 0.05).

b Highly significant difference ( *p* ≤ 0.01).

### AFM 3D Surface Topography Images

3D surface topography images were generated by AFM. The images show the top view of the samples, with the depth along the *z* -axis represented by different colors. Dark areas represent the pores, and light areas represent the peaks. Each type of aligner 3D image revealed unique and heterogeneous surface characteristics of the studied aligner samples, which exhibited pitting, dimples, grooves, or striations with varying heights and depths. TF samples displayed uniform surfaces with sharp triangular projections and few pores ( Fig. 1 ). DP samples are distinguished primarily by linear and rounded projections, pores, uneven regions, and narrow-deep scratch lines ( Fig. 2 ). Features became more pronounced after cleaning and *in vitro* aging.

**Fig. 1:**
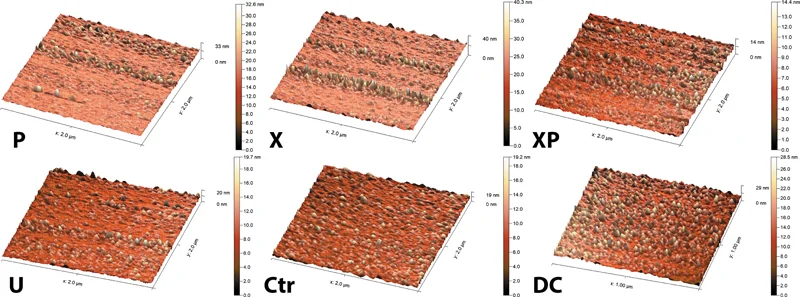
Atomic force microscopy (AFM) three-dimensional (3D) topographic images of thermoformed aligners, showing the as-received state and the effects of cleaning protocols (scan size: 2 µm × 2 µm). The *z* -axis shows surface height (light regions: peaks; dark regions: valleys/pores). Heterogeneous surface morphology is evident, with features including pitting, dimples, grooves, and striations (P, Polident cleaning group; X, brushing group; XP, brushing + Pril group; U, ultrasonic cleaning group; Ctr, control group; DC, as-received group).

**Fig. 2:**
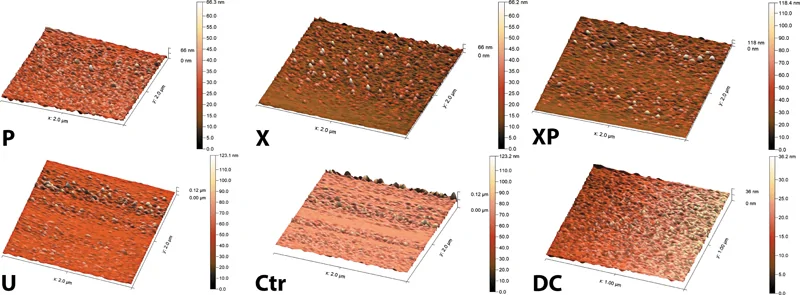
Atomic force microscopy (AFM) three-dimensional (3D) topographic images of 3D-printed aligners, showing the as-received state and the effects of cleaning protocols (scan size: 2 µm × 2 µm). The *z* -axis shows surface height (light regions: peaks; dark regions: valleys/pores). Heterogeneous surface morphology is evident, with features including pitting, dimples, grooves, and striations (P, Polident cleaning group; X, brushing group; XP, brushing + Pril group; U, ultrasonic cleaning group; Ctr, control group; DC, as-received group).

## Discussion

Patient compliance and behaviors must be considered when using CAs or esthetic brackets, such as ceramic or sapphire brackets. These orthodontic appliances are susceptible to staining from mouthwashes or chromogenic substances. Patients must follow the orthodontist's recommendations to use cleaning agents and limit the intake of staining substances throughout the orthodontic treatment period. 13 14 26

This study evaluated the effects of various cleaning protocols on the optical properties (T%) and surface roughness (Sa) of TF and DP orthodontic aligners after *in vitro* aging. Significant material-dependent differences and the observed effects of *in vitro* aging led to the partial rejection of the null hypothesis. The study groups were designed to enable a systematic comparison between TF and 3D-printed aligners, representing the two main manufacturing approaches currently used in clinical orthodontics. For each material type, the application of different cleaning protocols revealed how routine maintenance methods may differentially affect surface and optical properties after aging. This grouping strategy allowed for a controlled and clinically relevant evaluation of material behavior.

Across all tested conditions, the TF aligners consistently presented higher transmittance (T%) values than their DP counterparts, indicating superior optical clarity. Furthermore, the greater optical degradation of the DP aligners may be due to the surface porosity and layer lines introduced during fabrication. 11 The additive manufacturing process of resins is prone to surface damage resulting from the accumulation of microscopic ridges and pores during printing. Surface degradation may impact comfort, esthetics, hygiene, and bacterial accumulation. On the other hand, water absorption can deteriorate the fit, which could lead to a decrease in patient compliance and the effectiveness of the therapy. Additionally, surface abrasion modifies the light reflection and scattering characteristics, which may reduce optical clarity. 18 27

The highest T% for both TF and DP aligners was observed in the as-received group. In contrast, the lowest T% for TF aligners was observed in the ultrasonic cleaning group, contradicting reports that ultrasonic cleaning devices enhance light transmittance in rough copolyester materials. 28 For DP aligners, the lowest T% was recorded in the control group, suggesting that aging markedly reduced translucency. This aligns with Wible et al, 29 who reported aging-related optical degradation in copolyester materials.

Post hoc Tukey's HSD tests revealed that, for TF aligners, the as-received group differed significantly from all other groups, except for the brushing + Pril group. This finding is consistent with Azmuddin et al, 30 who reported that Dawn dish soap caused minimal physical alterations to polypropylene polymers. The ultrasonic cleaning group exhibited significant differences from all other groups, except the control group, suggesting that alternative cleaning protocols may better preserve optical clarity. The as-received group exhibited significant differences from all other groups in relation to DP aligners. Moreover, the brushing + distilled water group differed significantly from all other groups, except the brushing + Pril group. This supports the findings of Šimunović et al that surface abrasion affects light reflection and scattering, consequently diminishing optical clarity when brushing CAs. 18

In terms of surface roughness, DP aligners exhibited significantly higher Sa values than TF aligners after exposure to cleaning methods, except in the ultrasonic cleaning group. This aligns with the findings of Koletsi et al, 31 who reported that DP aligners demonstrated significantly greater surface roughness than TF aligners. TF aligners demonstrate reduced water absorption and enhanced dimensional stability due to their smooth surfaces and production methods. 11 24

The as-received group exhibited the highest mean nanoroughness (Sa) values, indicating that the cleaning protocols reduced the surface roughness of TF samples. Variations in additive manufacturing processes, including printing parameters (e.g., build orientation, layer thickness, and type of 3D printing technology) and postprocessing steps (e.g., curing time, equipment, postcuring temperature, and resin removal by centrifugation), may compromise fabrication consistency and accuracy, leading to nonuniform or noncomparable surface characteristics. 31 3D-printed aligners may have a rougher surface when built at a 45-degree angle, in line with a recent study by Wu et al. 32 The orientation of the build can markedly affect the stainability characteristics of 3D-printed aligners. 27 Meanwhile, a print layer thickness of 50 µm could minimize the stair-step effect and improve surface smoothness. It was found that the samples manufactured using liquid crystal display technology exhibited higher surface roughness, thereby compromising their final transparency. 33 A centrifuge cleaning method was employed instead of isopropyl alcohol to produce more translucent aligners. 34 Then, printed samples were UV-cured under a nitrogen atmosphere using a THC 2 system to mitigate oxygen inhibition by displacing ambient oxygen. 10 The polyurethane base of the TC 85 resin is susceptible to staining agents. 35 Textured surfaces exhibit increased interactions with dyes, facilitating pigment accumulation. 9 13 Fractures, scratches, and porosities in the material create niches for bacterial invasion and facilitate the absorption of saliva components. 24

The Polident tablets group showed the lowest Sa values, indicating its suitability for cleaning TF aligners. However, Agarwal et al 36 reported that Polident tablets did not affect the surface roughness of the polyurethane specimens. For DP samples, the highest Sa values were recorded in the control group, consistent with the findings of Koletsi et al, 31 who observed greater surface irregularities and reduced smoothness in DP aligners after intraoral aging. Thermocycling simulates aging by exposing aligner samples to alternating temperatures during water storage, resulting in increased water solubility and absorption, reduced transparency, and increased surface roughness. 23 33 37

The lowest Sa values were found in the ultrasonic cleaning group, indicating its suitability for cleaning DP aligners. This finding supports those of Susarchick et al, 28 who reported that aligner surface roughness significantly influences the effectiveness of cleaning solutions in stain removal. They noted that ultrasonic cleaning had a more pronounced effect on rough specimens by enhancing light transmittance.

Games–Howell post hoc tests for TF aligners revealed a statistically significant difference between the Polident tablet and brushing + distilled water groups. This aligns with findings that brushing can affect the surface properties of orthodontic materials 38 and supports the findings of Šimunović et al, 18 who demonstrated that mechanical cleaning with a toothbrush increases surface roughness, potentially compromising material integrity. In contrast, chemical cleaning with effervescent tablets resulted in smoother surfaces but led to material degradation over time. These tablets dissolve contaminants without mechanical damage, thereby reducing bacterial concentrations and maintaining smoother surfaces. Analysis of water absorption indicated that polyurethane aligners exhibit dimensional changes when immersed in saline and chemical solutions, which may affect their clinical performance. 18

Tukey's HSD post hoc tests for DP aligners revealed significant differences between the brushing + Pril and control groups. This aligns with findings that mechanical cleaning with a toothbrush and an additional agent, such as Pril dish soap, is effective for debris removal. While toothpaste is often discouraged because of its abrasive components, which can scratch CA surfaces and promote microbial colonization, Pril dish soap resulted in lower roughness and is therefore recommended for cleaning DP aligners. 30

The mean nanoroughness values (Sa) of the studied samples did not exceed 0.2 µm, regardless of cleaning protocol. From a clinical perspective, a previous study suggests that dental materials should have a surface roughness below 0.2 µm to be acceptable, as rougher surfaces increase plaque retention. Furthermore, a roughness value greater than 0.5 µm on an intraoral hard surface may cause discomfort and be perceptible to the patient's tongue. 15 Patients may become aware of changes in T% when they exceed approximately 5%. 39

AFM offers several advantages over scanning electron microscopy, including superior resolution, the ability to generate 3D surface profiles, and the provision of quantitative nanoscale surface roughness data. Additionally, surface topography parameters obtained from AFM are considered to be more representative of the true surface morphology. 7 15 18

Future *in vivo* research is required to assess the material properties, such as mechanical, thermal, and chemical characteristics, for different aligner materials and cleaning agents.

### Strengths and Limitations

The main strength of this study is its direct comparison of TF and 3D-printed aligners subjected to different cleaning protocols under standardized aging conditions (thermocycling and artificial saliva). The use of 3D-shaped aligners, rather than flat specimens, also provides a more clinically relevant simulation for assessing optical and morphological changes. However, the study is limited by its *in vitro* design, which does not fully replicate the complexity of intraoral conditions, including chemical erosion, blood contamination, biofilm formation, pH fluctuations, and mechanical stress from variable occlusal forces. In addition, only a small number of materials were evaluated.

### Generalizability

Only one brand of TF and 3D-printed aligners was tested; therefore, the results may not be fully generalizable to other materials, manufacturing processes, or postprocessing protocols.

## Conclusion

Within the limitations of this *in vitro* study, the manufacturing technique, aging process, and cleaning protocol significantly influenced the surface morphology and optical clarity of the CAs. TF aligners demonstrated greater translucency and smoother surfaces than their DP counterparts, justifying their clinical preference when esthetics and hygiene are prioritized.
